# Why suppression of HIV replication does not always make everything better

**DOI:** 10.1186/1758-2652-13-S4-K1

**Published:** 2010-11-08

**Authors:** M Lederman

**Affiliations:** 1 Case Western Reserver University, University Hospitals/Case Medical Center, Cleveland, USA

## 

The advent of combination antiretroviral therapies has altered the clinical course of HIV infection such that successful suppression of HIV replication typically results in sufficient restoration of immune function to protect most patients from the opportunistic complications that have defined AIDS. Yet a substantial proportion of treated HIV infected persons fail to increase circulating CD4 T cell counts to "normal" levels and a failure of CD4 T cell restoration predicts increased an increased risk of life threatening events that include malignant, hepatic and cardiovascular morbidities. Recent work suggests that the drivers of immune deficiency and "non-AIDS" complications of HIV infection may be intimately linked and in both settings, immune activation and inflammation are central. To this end, we have accused both HIV and the translocation of microbial elements from the damaged gut as drivers of both progressive CD4 T cell depletion and an increased tendency to coagulation and thrombosis formation in HIV infection. The Cleveland Immune Failure (CLIF) study was designed to explore the characteristics and potential drivers of disease pathogenesis among HIV infected persons who failed to achieve robust CD4 T cell increases despite successful suppression of HIV replication with combination antiretroviral therapy. In this study, we find evidence that immune activation, inflammation and increased thrombosis persist even in the setting of "complete" control of HIV replication and propose that this is not related to ongoing HIV replication but more likely to translocation of microbial products from the damaged gut.

